# A Global View of the Oncogenic Landscape in Nasopharyngeal Carcinoma: An Integrated Analysis at the Genetic and Expression Levels

**DOI:** 10.1371/journal.pone.0041055

**Published:** 2012-07-17

**Authors:** Chunfang Hu, Wenbin Wei, Xiaoyi Chen, Ciaran B. Woodman, Yunhong Yao, John M. Nicholls, Irène Joab, Sim K. Sihota, Jian-Yong Shao, K. Dalia Derkaoui, Aicha Amari, Stephanie L. Maloney, Andrew I. Bell, Paul G. Murray, Christopher W. Dawson, Lawrence S. Young, John R. Arrand

**Affiliations:** 1 School of Cancer Sciences, University of Birmingham, Birmingham, United Kingdom; 2 Department of Pathology, Guangdong Medical College, Zhanjiang, Guangdong, China; 3 Department of Pathology, University of Hong Kong, Hong Kong, China; 4 UMR542 Inserm-Université Paris Sud, Villejuif, France; 5 Department of Molecular Diagnostics, Sun Yat-Sen University Cancer Centre, Guangzhou, China; 6 Laboratoire de Biologie du Développement et de la Différenciation, Faculté des Sciences, Université d’Oran, Oran, Algeria; 7 ORL Centre Hospitalier et Universitaire, Oran, Algeria; The Chinese University of Hong Kong, Hong Kong

## Abstract

Previous studies have reported that the tumour cells of nasopharyngeal carcinoma (NPC) exhibit recurrent chromosome abnormalities. These genetic changes are broadly assumed to lead to changes in gene expression which are important for the pathogenesis of this tumour. However, this assumption has yet to be formally tested at a global level. Therefore a genome wide analysis of chromosome copy number and gene expression was performed in tumour cells micro-dissected from the same NPC biopsies. Cellular tumour suppressor and tumour-promoting genes (TSG, TPG) and Epstein-Barr Virus (EBV)-encoded oncogenes were examined. The EBV-encoded genome maintenance protein EBNA1, along with the putative oncogenes LMP1, LMP2 and BARF1 were expressed in the majority of NPCs that were analysed. Significant downregulation of expression in an average of 76 cellular TSGs per tumour was found, whilst a per-tumour average of 88 significantly upregulated, TPGs occurred. The expression of around 60% of putative TPGs and TSGs was both up-and down-regulated in different types of cancer, suggesting that the simplistic classification of genes as TSGs or TPGs may not be entirely appropriate and that the concept of context-dependent onco-suppressors may be more extensive than previously recognised. No significant enrichment of TPGs within regions of frequent genomic gain was seen but TSGs were significantly enriched within regions of frequent genomic loss. It is suggested that loss of the FHIT gene may be a driver of NPC tumourigenesis. Notwithstanding the association of TSGs with regions of genomic loss, on a gene by gene basis and excepting homozygous deletions and high-level amplification, there is very little correlation between chromosomal copy number aberrations and expression levels of TSGs and TPGs in NPC.

## Introduction

Nasopharyngeal carcinoma (NPC) is a highly malignant tumour of the post-nasal space. It is histologically heterogeneous, often containing substantial numbers of tumour-infiltrating lymphocytes [1] and has a curious aetiology with geographical, inherited, environmental and viral components [2]. Although the incidence is less than 1 per 100,000 of population per year throughout most of the world, in parts of Southeast Asia it reaches up to 30 per 100,000. Southern Italy, Greece, Turkey and the Maghreb region of North Africa have an intermediate incidence of about 8 per 100,000. Heritable cofactors include HLA haplotype and several genetic susceptibility loci whilst proposed environmental associations include carcinogenic nitrosamines present in some ethnic foods of high-and intermediate-risk regions [3], [4] and indigenous plants that contain activators of Epstein-Barr virus (EBV), the viral component of NPC aetiology [5].

In common with other cancers, NPC tumour cells carry various chromosomal abnormalities. Studies using conventional and array-based comparative genomic hybridisation (CGH), (collated in [6], [7]), have localised regions of chromosomal gain and loss. Although there are several reports of global analysis of gene expression in NPC [8]–[15], none of the previous studies has examined both chromosomal aberrations and gene expression changes in the same samples. It is believed that in the process of carcinogenesis, chromosomal gains and losses are linked to the activation or repression of oncogenes and tumour suppressors. In this study genetic copy number changes were examined in the context of alterations in the expression of tumour-promoting and tumour-suppressing genes (TPGs, TSGs) using a collection of NPCs that were obtained from high-and intermediate-incidence areas. Differential expression of a large number of genes that have previously been suggested as being tumour-promoting or tumour-suppressing was observed. However the differential regulation of many of these was not consistent with their previously proposed role and reinforces the concept of onco-suppressors and the context dependence of tumour suppressors and promoters.

Regions of the genome that showed frequent copy number aberrations were identified. Genes previously reported as tumour suppressors were significantly associated with regions of frequent genomic loss whereas putative tumour promoting genes were not enriched within regions of gain. Counter-intuitively, there was very little correlation between genomic copy number changes and levels of expression of TPGs and TSGs.

## Results and Discussion

### EBV Status and Latent Gene Transcription

The oncogenic human herpesvirus EBV is closely associated with NPC [5]. To assess the EBV status of the samples used in this study, the presence of EBV genomes in total cellular DNA and/or the expression of EBV-specific transcripts was determined (Table 1, Figure S1). 15 NPC biopsies and cell line C666-1 were examined for the presence of EBV DNA. All except tumour MMAH were found to be EBV-positive.

**Table 1:** Samples and their properties.

Sample Designation	Origin	Patient Ethnicity	Gender	EBVDNAStatus	EBNA1	BARF1	LMP1	LMP2
XY5	Guangdong	Cantonese	M	+	ND	ND	ND	ND
XY6	Guangdong	Cantonese	F	+	+	−	−	−
XY8	Guangdong	Cantonese	M	+	+	+	+	+
XY23	Guangdong	Cantonese	M	+	ND	ND	ND	ND
HKC1	Hong Kong	Cantonese	F	+	ND	ND	ND	ND
HKD1	Hong Kong	Cantonese	M	+	+	−	+	+
HK4	Hong Kong	Cantonese	M	+	+	+	+	+
HK6	Hong Kong	Cantonese	M	+	ND	ND	ND	ND
MDIG	France	Italian	M	+	+	+	+	+
MKAV	France	Italian	M	+	+	+	+	+
MKEC	France	Maghreb	M	+	+	+	+	+
MMAH	France	Maghreb	F	−	−	−	−	−
MOUZ	Algeria	Algerian	F	+	−	+	+	+
C666-1 cell line	Hong Kong	Cantonese	M	+	+	+	+	+
**SNP array only**								
XY16	Guangdong	Cantonese	M	+	+	+	+	+
HKC2	Hong Kong	Cantonese	F	+	ND	ND	ND	ND
**Expression array only**								
YH7	Guangdong	Cantonese	F	ND	+	−	+	+
YH8	Guangdong	Cantonese	M	ND	−	+	+	+
MSTA (normal)	Algeria	Algerian	U	ND	−	−	−	−
MBEZ (normal)	Algeria	Algerian	U	ND	−	−	−	−
MHAU (normal)	France	Italian	M	ND	−	−	−	−
T3 (normal)	UK	UK	U	ND	ND	ND	ND	ND

The names and properties of the samples are indicated, together with their EBV genome status (EBV DNA) and status of expression of the EBV genes EBNA1, BARF1, LMP1 and LMP2. Both expression array data and SNP array data were obtained from the first 13 biopsies and cell line C666-1. “SNP array only” indicates that only SNP array data were obtained from these biopsies whilst “Expression array only” signifies that only expression array data were obtained. U  =  unknown; ND  =  not determined.

The pattern of EBV gene expression in NPC is referred to as “Latency II” in which only EBNA1, the latent membrane proteins (LMPs), EBERs and transcripts from the BamHI-A region of the genome are expressed [5]. In addition, BARF1, a homologue of the human proto-oncogene c-fms [16], appears to be a latent gene in NPC [17]. Three of these genes are potential oncogenes in NPC: LMP1 has been referred to as the main transforming protein of EBV [5], LMP2 can transform epithelial cells *in vitro* [18] and BARF1 has oncogenic properties ([19] and refs therein). RNA from 12 NPC biopsies, 3 normal samples and cell line C666-1 were assayed for the expression of transcripts for EBNA1 and the three putative oncoproteins LMP1, LMP2 and BARF1. LMP1, LMP2 and BARF1 transcripts were detected in 11/13 (85%), 11/13 (85%) and 9/13 (69%) tumour samples (including C666-1) respectively. No EBV-specific transcription was detected in the normal samples suggesting that they are indeed EBV-negative, or in tumour MMAH, consistent with its apparent EBV DNA-negativity. Curiously, although other EBV-specific transcripts were detected in samples MOUZ (shown to be EBV DNA-positive) and YH8, EBNA1 transcripts were not seen. The basis of this unexpected result was not pursued but could be due to sequence variation leading to inefficient pcr primer binding or the highly repetitive, GC-rich sequences in the EBNA1 mRNA interfering with the amplification of this message in these two samples.

Although tumour MMAH had the histological characteristics of NPC, was diagnosed as such by at least two pathologists and had an overall gene expression profile that clustered with EBV-positive NPCs (data not shown), we were unable to confirm its EBV-positivity. It is possible that this is a rare case of an EBV-negative, non-keratinising NPC.

### Cellular Gene Expression Analysis

Expression array analysis of cellular gene expression levels was carried out using RNA from tumour cells of 15 NPC biopsies of various ethnic origin and NPC cell line C666-1 (hereafter collectively referred to as “tumours”) compared to four samples of normal epithelia (Table 1). The extent of relatedness of the overall gene expression profiles between the samples was examined by correlation analysis. This indicated that the expression profiles of tumours from different ethnic origins were closely related to each other but quite distinct from that of the normal samples.

Comparison of the gene expression of tumour cells versus normal controls also revealed that the Wnt, TGF-beta and Hedgehog signalling pathways were dysregulated. These observations agree with and extend those in earlier NPC gene expression studies [8], [9], [10] and will be presented in full elsewhere. Additional genome-wide expression studies of NPC have focused attention on other signalling pathways [14], MHC class I [10], cell cycle regulation [15], DNA repair and nitrosamine metabolism [9] or a single TSG [13]. The current analysis concentrates on genes that have been proposed to have a role in oncogenesis (e.g. oncogenes, tumour suppressor genes) and identifies a number of such differentially expressed genes that have not previously been implicated in NPC. Some of these have been identified but not discussed in the earlier studies. They are noted in Tables S1 and S2.

### Differential Expression of Tumour-related Genes

**Figure 1:**
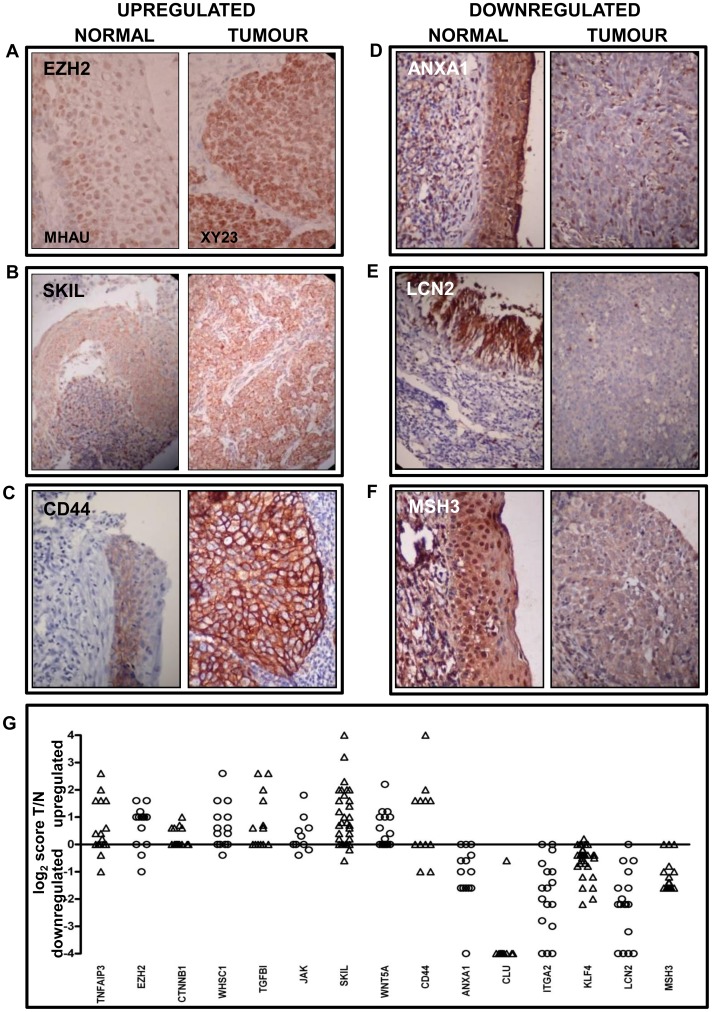
Immunohistochemical validation of differential regulation. Panels A–F show normal epithelium on the left and tumour tissue on the right. Panel A uses frozen sections from the same samples that were used in the array analysis (MHAU; normal epithelium: XY23; NPC), Panels B–F are paired specimens from the NPC tissue array. A–C: the upregulated genes EZH2, SKIL and CD44. D–F: the downregulated genes ANXA1, LCN2 and MSH3. Panel G summarises all the tissue array staining. The Y axis shows the log_2_ value of the ratio of the paired tumour:normal IHC scores. Some IHC scores were zero resulting in log_2_ ratio values of plus or minus infinity. For convenience, these are represented as 4 or−4 on the figure. Except for JAK and CD44, p values were less then 0.05. Individual p values are listed in Tables S1 and S2.

The expression array data were examined for differential expression of 1049 *a priori* determined, tumour-related genes. These genes comprised 309 putative tumour-promoting genes (TPGs), (including oncogenes, apoptosis/anoikis-suppressing and metastasis-promoting genes) and 740 putative tumour suppressors (TSGs). Expression changes in a number of differentially expressed genes representing each of the above categories were confirmed at the protein level by immunohistochemistry (IHC). In a few cases the same samples that were used for array analysis were available but usually, because of sample limitations, an NPC tissue array constructed from a different sample set was used. The IHC validation results are presented in Figure 1 and Tables S1 and S2.

### Upregulated Genes

From the *a priori* list of 1049 putative tumour-related genes, 346 were found to be upregulated more than twofold in at least four (25%) tumours and include 124 that have previously been shown to be upregulated in NPC or implicated in its pathogenesis (Table S1). The mean number of TPGs upregulated in each sample was 89 (range 36–115). The top 48 putative TPGs that were upregulated in 12 or more (75%) samples are listed in Table 2. Most of these have not previously been implicated in NPC.

**Table 2:** *A* *priori* defined, putative tumour promoting genes upregulated more than twofold in at least 12 (75%) samples.

Gene Symbol	Description	No. ↑	No. NC	No. ↓
TFRC	transferrin receptor (p90, CD71)	16	0	0
ITGAV	integrin, alpha V (vitronectin receptor, alpha polypeptide, antigen CD51)	16	0	0
BCL2	B-cell CLL/lymphoma 2	16	0	0
ROBO1	roundabout, axon guidance receptor, homolog 1 (Drosophila)	16	0	0
NCOA3	nuclear receptor coactivator 3	15	1	0
ETS1	v-ets erythroblastosis virus E26 oncogene homolog 1 (avian)	15	1	0
TNFAIP3	tumor necrosis factor, alpha-induced protein 3	15	1	0
RAN	RAN, member RAS oncogene family	14	2	0
EIF4A2	eukaryotic translation initiation factor 4A, isoform 2	14	2	0
FUS	fusion (involved in t(12;16) in malignant liposarcoma)	14	2	0
TPR	translocated promoter region (to activated MET oncogene)	14	1	1
JAK2	Janus kinase 2 (a protein tyrosine kinase)	14	2	0
RHEB	Ras homolog enriched in brain	14	2	0
PDE4DIP	phosphodiesterase 4D interacting protein (myomegalin)	14	2	0
RAB18	RAB18, member RAS oncogene family	14	2	0
GNA13	guanine nucleotide binding protein (G protein), alpha 13	14	2	0
ASAP1	development and differentiation enhancing factor 1	14	2	0
XIAP	baculoviral IAP repeat-containing 4	14	2	0
BIRC3	baculoviral IAP repeat-containing 3	14	2	0
SPARC	secreted protein, acidic, cysteine-rich (osteonectin)	14	2	0
RBM15	RNA binding motif protein 15	13	3	0
RAP1B	RAP1B, member of RAS oncogene family	13	3	0
DEK	DEK oncogene (DNA binding)	13	3	0
EZH2	enhancer of zeste homolog 2 (Drosophila)	13	3	0
LCP1	lymphocyte cytosolic protein 1 (L-plastin)	13	3	0
TOP1	topoisomerase (DNA) I	13	2	1
WHSC1	Wolf-Hirschhorn syndrome candidate 1	13	3	0
RAB28	RAB28, member RAS oncogene family	13	3	0
AKT3	v-akt murine thymoma viral oncogene homolog 3 (protein kinase B, gamma)	13	3	0
RPL22	ribosomal protein L22	13	3	0
RAP2C	RAP2C, member of RAS oncogene family	13	3	0
ECT2	epithelial cell transforming sequence 2 oncogene	13	3	0
TPM3	tropomyosin 3	13	3	0
NRAS	neuroblastoma RAS viral (v-ras) oncogene homolog	13	3	0
KAT6A	K(lysine) acetyltransferase 6A	13	3	0
KDSR	3-ketodihydrosphingosine reductase	13	3	0
NTRK2	neurotrophic tyrosine kinase, receptor, type 2	13	3	0
CTNNB1	catenin (cadherin-associated protein), beta 1, 88 kDa	13	3	0
ATF2	activating transcription factor 2	13	3	0
CLTC	clathrin, heavy chain (Hc)	12	3	1
JUN	jun oncogene	12	4	0
RAP1A	RAP1A, member of RAS oncogene family	12	4	0
TRIM24	tripartite motif-containing 24	12	4	0
CIITA	class II, major histocompatibility complex, transactivator	12	4	0
PSIP1	PC4 and SFRS1 interacting protein 1	12	4	0
PICALM	phosphatidylinositol binding clathrin assembly protein	12	4	0
SKIL	SKI-like oncogene	12	4	0
ITGB6	integrin, beta 6	12	4	0

↓  =  downregulated, ↑  =  upregulated, NC  =  no change. Further information can be found in Table S1.

Frozen sections taken from biopsies that were used for the array analysis were used in IHC to confirm upregulation of the oncogene EZH2 (Figure 1A). EZH2 expression was also validated using an NPC tissue array, along with the TPG SKIL (Figure 1B).

NPC is highly metastatic, with 75% of metastases occurring in bone. Upregulated, bone metastasis-associated genes include *NOV* and *TNFRSF11A* which were upregulated in 10 and 5 tumours, respectively (Table S1). The metastasis-associated gene osteopontin (*SPP1*), a target of aberrant Wnt signalling that has been implicated in NPC was upregulated in 11 tumours (Table S1). Immunohistochemical staining validated upregulation of the metastasis-associated, TGFβ pathway target, TGFBI (Figure 1G).

Upregulated antiapoptotic genes include the NPC-associated genes *BIRC3, BCL2* and *CLDN1* which is also a target of the Wnt signalling pathway. Upregulation of the anti-apoptotic gene TNFAIP3 was confirmed at the protein level (Figure 1G).

Anoikis is a form of apoptosis that is induced by loss of, or inappropriate, cell adhesion. A variety of genes, including the Wnt pathway-associated *CTNNB1* (Figure 1G), that have been implicated in mechanisms of anoikis resistance were found to be upregulated in many tumour samples.

### Downregulated Genes

140 genes from the *a priori* list of tumour-related genes were downregulated more than twofold in four or more tumours and include 40 that have been independently reported to be downregulated in NPC (Table S2). 115 of these genes have been proposed to be TSGs in other contexts, including 7 in NPC. The mean number of TSGs downregulated in each tumour was 76 (range 56–93). 52 putative TSGs, the majority of which have not been previously implicated in NPC, were downregulated in 12 or more (75%) samples (Table 3).

**Table 3:** *A priori* defined, putative tumour suppressor genes downregulated more than twofold in at least 12 (75%) samples.

Gene Symbol	Description	No. ↓	No. NC	No. ↑
CEACAM1	carcinoembryonic antigen-related cell adhesion molecule 1 (biliary glycoprotein)	16	0	0
CLCA2	chloride channel, calcium activated, family member 2	16	0	0
CLU	clusterin	16	0	0
DUOX2	dual oxidase 2	16	0	0
EHF	ets homologous factor	16	0	0
EPAS1	endothelial PAS domain protein 1	16	0	0
H19	H19, imprinted maternally expressed untranslated mRNA	16	0	0
JUP	junction plakoglobin	16	0	0
KLK11	kallikrein-related peptidase 11	16	0	0
LCN2	lipocalin 2 (oncogene 24p3)	16	0	0
MSMB	microseminoprotein, beta–	16	0	0
MSRA	methionine sulfoxide reductase A	16	0	0
PER2	period homolog 2 (Drosophila)	16	0	0
S100A2	S100 calcium binding protein A2	16	0	0
SOX7	SRY (sex determining region Y)-box 7	16	0	0
VWA5A	von Willebrand factor A domain containing 5A	16	0	0
ZNF185	zinc finger protein 185 (LIM domain)	16	0	0
RAB25	RAB25, member RAS oncogene family	16	0	0
SFN	stratifin	15	1	0
ANXA1	annexin A1	15	1	0
DLG1	discs, large homolog 1 (Drosophila)	15	1	0
DUOX1	dual oxidase 1	15	1	0
GJB2	gap junction protein, beta 2, 26 kDa	15	1	0
GPX3	glutathione peroxidase 3 (plasma)	15	1	0
KLF5	Kruppel-like factor 5 (intestinal)	15	1	0
SERPINB13	serpin peptidase inhibitor, clade B (ovalbumin), member 13	15	1	0
TACC2	transforming, acidic coiled-coil containing protein 2	15	1	0
ING2	inhibitor of growth family, member 2	14	2	0
BRD7	bromodomain containing 7	14	2	0
IGFBP5	insulin-like growth factor binding protein 5	14	2	0
PERP	PERP, TP53 apoptosis effector	14	1	1
SDHC	succinate dehydrogenase complex, subunit C, integral membrane protein, 15 kDa	14	2	0
SERPINB2	serpin peptidase inhibitor, clade B (ovalbumin), member 2	14	1	1
SLC9A3R1	solute carrier family 9 (sodium/hydrogen exchanger), member 3 regulator 1	14	2	0
CSK	c-src tyrosine kinase	13	3	0
LLGL2	lethal giant larvae homolog 2 (Drosophila)	13	3	0
RARB	retinoic acid receptor, beta	13	3	0
RPS6KA2	ribosomal protein S6 kinase, 90 kDa, polypeptide 2	13	3	0
SEMA3F	sema domain, immunoglobulin domain (Ig), short basic domain, secreted, (semaphorin) 3F	13	3	0
CBFA2T3	core-binding factor, runt domain, alpha subunit 2; translocated to, 3	12	4	0
CEBPD	CCAAT/enhancer binding protein (C/EBP), delta	12	4	0
CXCL14	chemokine (C-X-C motif) ligand 14	12	2	2
GLTSCR2	glioma tumor suppressor candidate region gene 2	12	4	0
HRASLS	HRAS-like suppressor	12	3	1
PDCD4	programmed cell death 4 (neoplastic transformation inhibitor)	12	3	1
PPP1R13B	protein phosphatase 1, regulatory (inhibitor) subunit 13B	12	4	0
PRDX2	peroxiredoxin 2	12	4	0
PYCARD	PYD and CARD domain containing	12	4	0
TMSB10	thymosin, beta 10	12	4	0
VTA1	Vps20-associated 1 homolog (S. cerevisiae)	12	4	0
WASL	Wiskott-Aldrich syndrome-like	12	4	0

↓  =  downregulated, ↑  =  upregulated, NC  =  no change. Further information can be found in Table S2.

Confirmation of downregulation of the TSGs ANXA1, LCN2, KLF4, CLU and MSH3 was obtained by immunohistochemistry (Figure 1D–G). Downregulation of ITGA2, which has been associated with tumour progression, was also verified. Although the expression studies found 115 previously characterised TSGs to be downregulated, even this number is an underestimate. Several NPC-associated TSGs, including *RASSF1* and *PTPRG* [20], [21] were expressed at low levels in both normal and tumour samples and were called “absent” by the analysis software. Thus relative expression levels could not be determined and these genes were not included in the list of downregulated genes. Similarly, other well-known NPC TSGs e.g. *CDKN2A* [22], *ATM* [13] and *ZMYND10* [23] were called “present” in only 3 normal samples and therefore did not fulfil the criteria to be designated as downregulated.

### Potential Onco-suppressor Genes

Examination of the expression data for the behaviour of specific genes in single tumours reinforces the concept of the individuality of each tumour. Although some TPGs and TSGs (e.g. *BCL2, VWA5A*) appear to be universally differentially expressed, the majority seem to be important in only a fraction of cases. In addition, although many putative TSGs and TPGs were respectively downregulated or upregulated in the tumour samples relative to normal tissue, a substantial number exhibited differential expression in the “wrong” direction. Previous studies have proposed that 208 of the 346 upregulated genes act as TSGs in other types of cancer. Other, independent studies have confirmed the upregulation of 127 (61%) of these genes in NPC or other tumour types (Table S1). Examples are *ROBO1*, *LATS2* and *SPARC*, which are tumour suppressive in several cancers [24]–[26] but upregulated and associated with tumour progression, metastasis and decreased survival in NPC [27]–[29]. Others include several Wnt-and TGFβ-pathway-associated genes and *MNX1, CLDN1, ATF2, SIRT1, PTPN13* and *E2F1* which have been discussed as possessing both tumour-promoting and tumour-suppressing properties (onco-suppressors) [30]–[35]. This suggests that, at least at the stage of tumour development when the samples were taken, these genes do not act as TSGs in NPC.

Similarly, 35 of the 115 downregulated, putative TSGs have been found to be upregulated in other tumour types. 6 of these (*CLU, LCN2, KLF4, KLF5, KLF6, SLC9A3R1*) have been discussed as onco-suppressors [36]–[41].

Of the 486 putative TPGs and TSGs that were identified as being differentially expressed, 273 (56%) appear to be both upregulated and downregulated in cancer. This is consistent with the increasing awareness that there exists a population of proteins that can act as either tumour suppressors or tumour promoters depending on cellular context such as tumour type, stage of tumour development or subcellular location. Similarly, the *TGFβ* signalling pathway has been shown to be either tumour promoting or suppressing [42]. We and others have shown this pathway to be dysregulated in NPC and several *TGFβ* pathway-associated putative TSGs show differential expression in the opposite direction to that expected from some literature reports. In this context, enhanced expression was observed of *PMEPA1*, which recently has been shown to be able to act as a molecular switch that converts TGFβ from a tumour suppressor to a tumour promoter [43].

Some putative TSGs and TPGs appeared to be upregulated in some samples whilst downregulated in others. Two such genes, the Wnt pathway-associated genes *WNT5A* and *CD44*, both of which on array analysis could not be regarded as predominantly up-or down-regulated, were examined by IHC (Figure 1C, G). WNT5A, which has been described as being either tumour promoting or suppressing [44], was upregulated at the protein level in 10/23 samples and unchanged in the remainder whilst CD44 protein expression was increased in 6/12, reduced in 2/12 and unchanged in the remaining 4 (Figure 1G). Similarly, the putative tumour suppressor protein, E-cadherin (CDH1) has been found to be both upregulated and downregulated in NPC [45]. On our expression arrays it appeared to be upregulated in 4 samples, downregulated in 3 and unchanged in 9. Whether such instances represent the particular gene product acting as a tumour suppressor in some cases, whilst being a promoter in others, is currently unknown.

Functional characterisation of putative TSGs or oncogenes using cell lines or animal models reveals dramatic effects on cell growth in response to the perturbation of expression of just a single TSG or oncogene (e.g. [20], [21]). Considering these data alongside the current observations that reveal dysregulation of large numbers of TSGs and TPGs per tumour, emphasises the magnitude of the loss of proliferative control in NPC and begs the question as to how many-or how few–aberrantly regulated TSGs and TPGs are required for tumourigenesis. It will be important to perform functional studies, in the appropriate context, of the putative TSGs and TPGs identified here and to confirm the apparent dual role of the putative onco-suppressor genes.

### Copy Number Changes in NPC Tumour Cells

**Figure 2:**
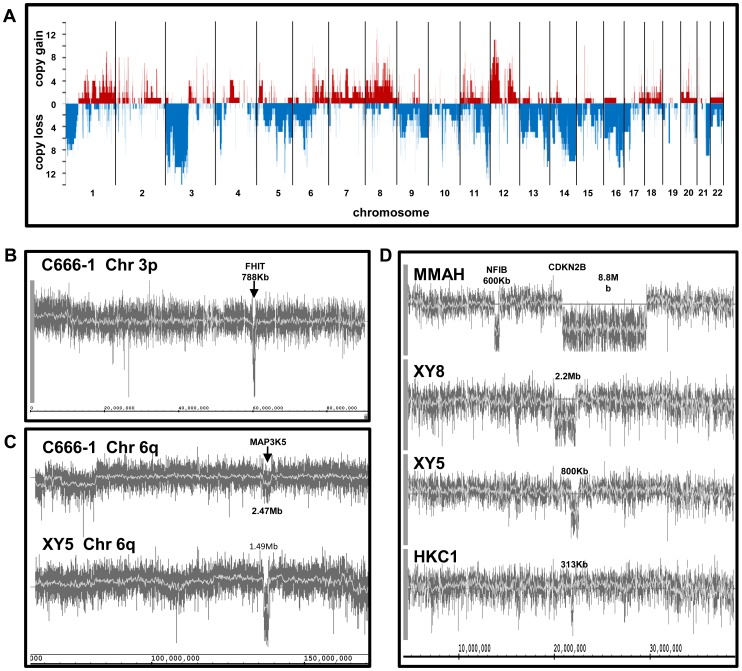
Chromosomal copy number changes. Panel A shows the major regions of copy number gain (red) or loss (blue) across the genome. The Y axis shows the number of cases (out of 16) at which a region was changed. Chromosomes are ordered from left to right as indicated. Panels B–D show traces of the log2 ratio of the copy number of DNA from the tumour samples compared to the normal controls. B: the homozygous deletion at the FHIT locus in C666-1; C: a hemizygous deletion in C666-1 and a homozygous deletion in tumour XY5, both at 6q22.33; D: homozygous deletions encompassing the CDKN2B locus in tumours MMAH, XY5, XY8 and HKC1. Tumour MMAH also shows a 600 Kb homozygous deletion at 9p24.1 containing the NFIB gene. The sizes of the discrete aberrations are indicated.

Analysis of amplified DNA from the C666-1 cell line and from 15 NPC biopsies revealed that all tumour samples showed extensive chromosomal copy number aberrations. The principal regions of loss were within 1p, 3p, 5q, 9p, 9q, 11q, 13, 14q, 16 and 21q whilst 8q, 12p and 12q showed the most significant gains (Figure 2A). These results are in broad agreement with data obtained in several previous studies [6], [7]. In particular, copy loss at 3p21.3, 9p21.3 and 11q23.3 was observed in 14/16 (88%), 9/16 (56%) and 11/16 (69%) of samples, respectively. The amplicon at 12p13.3 that was recently identified by Or et al [46] in 51% of primary tumours exhibited copy number gain in 4 (25%) tumours. Less consistent with earlier data, the 11q13.3 locus at which Hui et al [47] found copy number gain in 62% of primary NPCs showed gain in only 1 sample (6%).

### TSGs are Enriched within Regions of Loss

After segmentation, copy number information was available for 21204 named genes, of which about 3.4% (714/21204) are putative TSGs. It was first asked for each sample whether TSGs are more frequently found in the group of genes showing reduced copy number. 12/16 samples showed a significant enrichment (p<0.05). The enrichment of TSGs in the deleted genes was calculated with different frequency cut-off values. 4.2% of genes deleted in 5 or more samples were TSGs, increasing progressively to 44% within those deleted in 14 or more samples (Table 4). The TSGs enriched within genomic segments deleted in 12 or more (75%) samples are listed in Table 4.

**Table 4:** Enrichment of TSGs with increasing frequency of genomic deletion.

No. of samples with deleted genes in common	No. of deleted genes	No. of deleted TSGs	% TSGs in deleted genes	P value of binomial test	Deleted TSGs
5 or more	5543	233	4.2%	4.8×10^−4^	
6 or more	4045	172	4.3%	1.5×10^−3^	
7 or more	2770	132	4.8%	6.8×10^−5^	
8 or more	1966	101	5.1%	3.0×10^−5^	
9 or more	1386	80	5.8%	3.8×10^−6^	
10 or more	975	57	5.9%	6.0×10^−5^	
11 or more	553	36	6.5%	1.7×10^−4^	
12 or more	161	20	12.4%	6.0×10^−7^	FAM107A, FHIT, HHATL, PCBP4, RBM5, RPS14 and the 14 TSGs below
13 or more	39	14	35.9%	1.6×10^−11^	LRIG1, POU2F3, PRKCD, and the 11 TSGs below
14 or more	25	11	44.0%	1.8×10^−10^	CACNA2D2, CYB561D2, HYAL1,HYAL2, NAT6, NPRL2, RASSF1, SEMA3B, SEMA3F, TUSC2, ZMYND10

The frequency of TSGs within deleted genes increases progressively as the common deleted region(s) are found in increasing numbers of samples. The TSGs found in the most frequently deleted regions are listed in the column on the right.

Although TPGs were found within deleted genes, they were significantly enriched in only one sample (C666-1).

About 1.4% (299/21204) of named genes were TPGs. There was no significant enrichment of TPGs within copy-gain genes in any sample. The enrichment of TPGs in the copy-gain genes was also calculated with different frequency cut-off values. TPGs were not significantly enriched in the genes showing gain of copy number (Table S3).

Although TSGs were present within the group of genes showing copy number gain, they were not significantly enriched in any of the samples.

### Determination of Significant Regions of Copy Number Change

To search for non-random copy number aberrations that may play a role in tumourigenesis, a standard analysis of Genomic Identification of Significant Targets in Cancer (GISTIC) [48] was performed. In the deletion peaks, which include the well-known TSG-containing loci at 3p21.31 and 9p21.3, TSGs were significantly (p = 4.14×10^−6^) enriched to 6.2% (62/993). Importantly, 42 deletion peaks contained a total of 62 TSGs (Table S4). Of these 62 deleted TSGs, 16 were downregulated as determined by expression array analysis. The downregulation of one of these, CLU, was verified at the protein level by IHC (Figure 1G). Additionally, genes that have been implicated as NPC TSGs were found within these deletion peaks. These genes include *CDKN2A, ZMYND10, RASSF1, NDRG1, TACC2* and *CACNA2D2* (Table S4).

Conversely, the expression array data indicated that 16 of these deletion peak-associated putative TSGs appeared to be upregulated. Independent studies also suggest that of these, *CDH1, COL4A1, BUB1B, RUNX3* and *SOCS1* are upregulated in NPC (Table S2) whilst the expression of a further 5 has been reported to be enhanced in other cancers.

177 peaks of copy gain were identified. In these peaks, 1.2% (6/515) of genes are TPGs, which are not significantly (p = 0.73) enriched. 6 peaks of copy number gain contained 6 TPGs (Table S5), of which 4 (*ERBB4, RAB21, PSIP1, ZNF384*) appeared to be upregulated (Table S1).

The observation of upregulated expression of some of the TSGs that were associated with frequently deleted genomic segments could signify that these putative TSGs, if indeed they do act as such in NPC, may be active as suppressors at an early stage of carcinogenesis but later convert to a tumour-promoting role. However it is also possible that, in keeping with the two-hit hypothesis, the protein products of these transcripts may be non-functional or absent due to mutation.

### Homozygous Deletions

It is believed that homozygous deletions (HD) in tumours are particularly noteworthy for their potential to encompass tumour suppressor gene loci [49]. 205 genes appeared to be homozygously deleted. 55 of these were within deleted segments validated by q-pcr (Table S6). 56% (9/16) of the NPC samples have homozygously deleted TSGs including *FHIT* and *CDKN2B*, both of which have been implicated in NPC [50], [51]. The enrichment of TSGs in the homozygously deleted genes was calculated with different frequency cut-off values (Table S7). TSGs were shown to be significantly enriched in homozygously deleted genes. However, increasing the cut-off frequency does not significantly increase the enrichment of TSGs.

In agreement with previous observations in a variety of tumours including NPC, [51] the TSG *CDKN2B* was a frequent target of HD (Figure 2D). Additionally, it was striking that in one sample the only genetic loss within chromosome 3p was a discrete homozygous deletion of the *FHIT* gene (Figure 2). This locus was hemizygously deleted in a further 11 tumours. Studies using knockout mice revealed that animals in which the *FHIT* gene was hemizygously or homozygously deleted were “exquisitely sensitive” to nitrosamine-mediated carcinogenesis [52]. These observations together with those showing that loss of chromosome 3p occurs in “almost all” [53] cases of primary NPC and in premalignant lesions [54] together with epidemiological evidence that dietary nitrosamines predispose to the development of NPC [2]–[4] suggest that hemizygous or homozygous loss of *FHIT* may be a driver of NPC tumourigenesis.

### Amplification within Chromosome 8 is Associated with Increased Gene Expression

**Figure 3:**
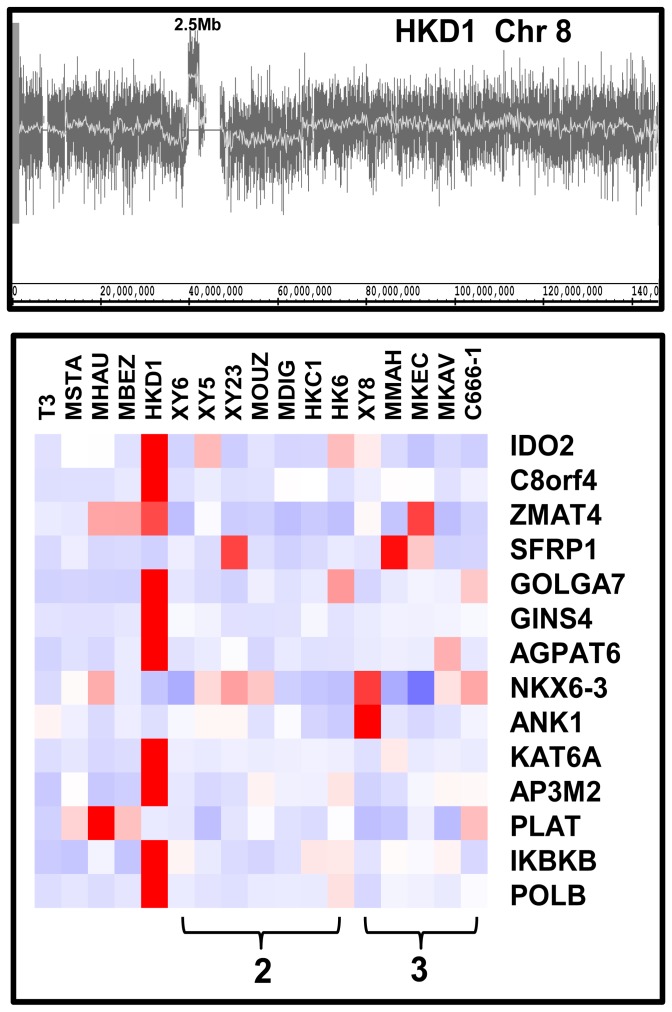
The 8p11.21 amplified region of tumour HKD1. A. Trace of the log2 ratio of the copy number of DNA from the tumour sample compared to the normal controls. The 2.5 Mb amplification is indicated. B. Heat map of the relative expression levels of the genes found within the amplified region. The samples appear in columns and the individual genes within the amplified region form the rows. High level expression is represented by the intensity of red and low level by blue. The brackets at the bottom indicate tumour samples with genome copy numbers of 2 or 3 within this region.

The only example of an amplification (5 or more copies) that was found in this sample set is a 2.5 Mb segment in tumour HKD1 (Figure 3). The array data suggested that this region contained 11.6 DNA copies whilst q-PCR analysis gave a value of 8.7 (Table S6). Expression arrays showed the mRNA levels of the majority of genes throughout this region, including the putative oncogene *KAT6A*, to be substantially upregulated in the amplified sample but relatively unchanged in samples with two or three copies (Figure 3). Relative to the mean of the normal samples, the expression of *KAT6A* in tumour HKD1 was upregulated 31-fold and was up more than twofold in 12 other tumours (Table 2). Interestingly, this amplified segment corresponds to the A4 amplicon defined in breast cancer where overexpression of the genes *KAT6A* and *AP3M2* was most significant [55]. However, the functional identity of any oncogene(s) in this region remains to be established.

### Expression Levels of TPGs and TSGs are Poorly Correlated with DNA Copy Number

It is frequently surmised that regions of chromosomal loss are likely to harbour (downregulated) tumour suppressor genes whilst regions of gain may be associated with overexpression of growth promoting genes. The frequency of putative TSGs encoded within deleted segments of the genome increased along with the proportion of samples containing a given deletion. This finding is in keeping with the theory of genomic loss being at least part of the mechanism of inactivation of TSGs in the process of tumourigenesis. Whilst the concept that gene expression changes in tumours are directly related to genomic copy number may be mechanistically appealing, in general, expression of the putative TSGs and TPGs in the NPC samples was not well-correlated with copy number. Expression and copy number data were available for 478 of them (Tables S1 and S2). Analysis of the level of gene expression versus DNA copy number on a case by case basis revealed that 469 of the 478 TPGs and TSGs (98.1%) had a Spearman’s correlation coefficient of less than 0.65.

This finding is similar to other studies, e.g. in glioma [56], which found that only a few genes within the total transcriptome showed good correlation between copy number and expression. It is increasingly apparent that reduction in expression of TSGs seems mostly to be achieved by epigenetic mechanisms such as promoter hypermethylation and histone deacetylation [57], [58] whilst upregulation of gene expression can be achieved by a variety of transcriptional regulatory mechanisms.

### Conclusions

This work is focussed on potential tumour-promoter and-suppressor genes in NPC. It shows that the EBV-encoded EBNA1 protein, and the three putative viral oncogenes LMP1, LMP2 and BARF1 are expressed in the majority of cases that were examined and also identifies a large number of potential cellular TPGs and TSGs, many of which have not previously been associated with NPC. A number of these were found to be components or targets of the Wnt and TGF-beta signalling pathways, providing evidence for dysregulation of these pathways in NPC. It shows that genes that have been described as potential tumour promoting genes are not significantly associated with genomic regions exhibiting gain of copy number. On the contrary, genes described as TSGs are significantly enriched within genomic regions that are frequently deleted even though the expression of some of these genes within hemizygous deletions appears to be upregulated. It is suggested that loss at the *FHIT* locus may be a driver of NPC tumourigenesis. Very little correlation is observed between the level of TPG and TSG expression and genomic copy number except for loss of expression in homozygous deletions and one highly amplified segment which shows enhanced gene expression. Individual NPC tumours each express a large number of dysregulated, putative, tumour-suppressing and tumour-promoting genes but almost 60% of these genes can be either upregulated or downregulated in different types of tumour. This suggests that the simplistic classification of genes as TSGs or TPGs may not be entirely appropriate and that the concept of onco-suppressors may be more extensive than previously recognised.

## Materials and Methods

### Ethics Statement

This study was approved by the South Birmingham Research Ethics Committee (Reference 09/H1207/95). All samples were anonymous.

The Paris samples were obtained between September 1992 and June 1995 using verbal consent in accordance with protocols (for the use of surgical tissues and medical records) previously approved by the local human studies committee.

The samples from Zhanjiang were obtained between 2001 and 2005 following local procedures applicable at that time.

The Hong Kong samples were obtained using verbal consent for donation of normal and tumour tissues for genetic research following a procedure approved by The Hong Kong University and Hospital Authority (Hong Kong West) Institutional Review Board (Reference UW 06-149 T/1174).

The samples from Oran, Algeria were obtained under authorisation from the senior medical management of the hospital and with the patients’ written consent.

One control sample came from a frozen section of a tonsil obtained, with informed written consent and ethical approval (South Birmingham Research Ethics Committee Reference 06/Q2702/50).

DNA from five individuals with normal DNA copy number was from blood samples taken with informed written consent and ethical approval (Cambridgeshire 3 Research Ethics Committee (Reference 09/H0306/79)). These samples had already been subjected to array-based DNA copy number analysis in the original study.

### NPC Biopsies

Snap-frozen biopsies of NPC were obtained from the Associated Hospitals of Guangdong Medical College, China; Queen Mary Hospital, Hong Kong; Institut Gustave Roussy, France and Oran Hospital, Algeria. All samples had concurrent formalin fixed material which was used and retained by local pathologists to diagnose the cases as NPC (undifferentiated, non-keratinising carcinoma). This diagnosis was confirmed by one of us (XC) on sections taken from the frozen tissue used in the analysis. Except for gender and ethnic origin, no further information was available. Samples were transported on dry ice and stored in liquid nitrogen until used. Tumour samples for analysis were selected on the basis of their having well-defined islands of tumour cells with minimal numbers of infiltrating lymphocytes. Three biopsies that did not contain any tumour tissue had areas of normal epithelium that were used as a source of control material for the expression studies. A fourth normal control came from a frozen section of a tonsil obtained from a UK patient. The samples and their origins are listed in Table 1. Samples were tested for EBV DNA-positivity by PCR on extracted DNA [59] and for the expression of EBV encoded genes by RT-PCR as described [60] using the primers listed in Table S8.

### NPC Tissue Array

An NPC tissue array consisting of paired samples containing both NPC tumour and adjacent nasopharyngeal mucosa was constructed using formalin-fixed, paraffin-embedded samples from the archives of the Pathology Department, Sun Yat-Sen University, Guangzhou [61]. EBER *in situ* hybridisation verified that all tumours were EBV-positive whereas the corresponding normal cells were negative.

### Immunohistochemistry

In a few cases the same samples that were used for array analysis were available but usually, because of sample limitations, an NPC tissue array constructed from a different sample set was used. FFPE tissue array sections were deparaffinised in xylene and rehydrated through ethanol to distilled water, then incubated with 3% hydrogen peroxide for 15 minutes to quench endogenous peroxidase. Antigen retrieval was performed using the agitated low temperature epitope retrieval method [62], or by heating in low pH retrieval buffer (Vector Laboratories) for 20 minutes at 880 W in a microwave oven. Sections were rinsed with PBS then incubated with primary antibody for one hour at room temperature. After three brief washes with PBS/Tween, slides were treated with a peroxidase-based secondary antibody (Dako EnVision™ Detection System, Denmark) for 30 minutes at room temperature. The final peroxidase-labelled complex was visualised using diaminobenzidine. The tissue sections were counterstained with hematoxylin, dehydrated, and mounted with coverslips.

For frozen tissue, 8 micron cryosections were cut, air dried and fixed in 10% formalin for 20 minutes. The sections were then incubated in 3% hydrogen peroxide for 15 minutes, followed by antigen retrieval in low pH buffer for 20 minutes as above. The subsequent procedures were as described for FFPE sections.

The antibodies used in IHC are listed in Table S9.

### IHC Scoring

A semiquantitative scoring system was used to evaluate IHC staining. Scores (values 0–9) were obtained by multiplying staining intensity (negative  = 0, weak  = 1, moderate  = 2, or strong  = 3) by the proportion of positive cells (≤30% = 1, 30%–70% = 2, >70% = 3).

### Cell Culture

The EBV-positive NPC cell line C666-1 [63] was obtained from the late Dolly Huang. Cells were cultured on fibronectin-coated dishes to ensure adherent cell growth. The EBV-positive B-lymphoblastoid cell line X50-7 [64] was provided by George Miller. Both C666-1 and X50/7 were cultured in RPMI medium 1640 supplemented with 10% FCS, 2 mM L-glutamine, and 1% penicillin–streptomycin solution (Sigma-Aldrich).

### Microdissection, Nucleic Acid Extraction and Amplification

8 micron cryosections were transferred onto PALM membrane slides (P.A.L.M. Microlaser Technologies) and air dried on ice for about 1 minute. Slides were immersed for 2 minutes in cold 75% ethanol, tapped dry and stained for 30 seconds in cold haematoxylin and eosin (9∶1) containing 1% NucleoGuard (AmpTec, Hamburg). Excess stain was tapped off and slides were washed in cold nuclease-free water for 30 seconds, cold 75% ethanol for 1 minute, cold 100% ethanol for 1 minute then air dried. Cells for analysis were excised by laser microdissection and pressure catapulting using a PALM MicroBeam instrument and caught on PALM Adhesive Caps. A minimum of 200,000 µm^2^ of tissue was collected for each DNA or RNA extraction. RNA was extracted by adding 100 µl of RLT buffer (Qiagen) supplemented with 1 µl of N-carrier (AmpTec) and 1 µl of NucleoGuard followed by incubation at room temperature for 15 minutes. Extracted RNA was cleaned up using a Qiagen RNeasy mini kit, including the on-column DNase step as per the manufacturer’s instructions. The eluted RNA was collected by ethanol precipitation in the presence of 1 µl P-carrier (AmpTec) and washed twice with 80% ethanol. After checking the quality using the pico assay on an Agilent Bioanalyser, RNA was subjected to three rounds of amplification followed by biotin labelling using an ExpressArt TR Nano amplification kit (AmpTec) and an Affymetrix IVT labelling kit as previously described [65].

Total RNA was extracted from C666-1 cells as described [65] and cleaned up as above. 20 ng was amplified and labelled as above.

DNA was extracted from microdissected cells by adding 100 µl of lysis buffer (10 mM Tris-HCl, pH 8.0, 1 mM EDTA, 1% Tween 20, 0.4 mg/ml proteinase K (Qiagen) to the tube containing the captured cells and incubating inverted for 3 hr at 55°C then 5 min at 95°C. 2 µl of linear polyacrylamide solution (GenElute, Sigma) were added and DNA was recovered by ethanol precipitation. The pellet was washed with 70% ethanol and air dried. The precipitated DNA was subject to whole genome amplification using a Genomiphi kit (GE Healthcare) according to the manufacturer’s instructions.

DNA was extracted from C666-1 and X50-7 cells using a DNeasy kit (Qiagen) according to the manufacturer’s instructions. DNA samples prepared from blood samples, taken from five individuals with normal DNA copy number, were a kind gift from Dr Tessa Webb.

### Hybridisation to Affymetrix Arrays

Biotinylated RNA was fragmented and hybridised to Affymetrix Human Genome U133Plus2 Arrays according to the Affymetrix protocol. C666 DNA and DNA amplified from cryosections were subjected to the Mapping 500 K Assay Protocol (Affymetrix) protocol.

All arrays were washed and stained on an Affymetrix FS450 fluidics station then scanned using an Affymetrix GeneChip 3000 7G scanner as per Affymetrix procedures. GCOS software (Affymetrix) was used for instrument control and data acquisition.

### Validation of Copy Number Analysis on Amplified DNA

The C666-1 cell line, an NPC-derived line that uniquely maintains the EBV genome in long term culture, was used to validate the SNP array copy number analysis and DNA amplification protocols. Amplified and unamplified C666-1 DNAs were analysed on 500 K arrays and showed a high degree of concordance. Genes identified as deleted using the amplified C666-1 DNA were over 99% identical with those identified using the unamplified DNA. Genes identified as being within regions of gain in the amplified C666-1 DNA were over 97% identical with those found using the unamplified DNA. In a total of 21204 named genes on somatic chromosomes, 99.53% (21105) were concordantly called deleted, gained or normal in the amplified and unamplified C666-1 samples.

### Array Data Analysis

Genotype analysis was performed using Affymetrix Genotyping Console version 4.0 with the default settings. QC call rates of the 44 arrays ranged from 81.7% to 95.2%. The array signal intensity CEL files of the NPC and amplified normal copy number control samples as well as 127 hapmap female samples (http://www.hapmap.org/downloads/raw_data/affy500k/) were analyzed together using dChip [66] with invariant set normalization and the PM/MM difference model. SNP-level raw log2 ratios relative to the average of the hapmap samples were exported from dChip and further analysed using R (http://www.r-project.org/). For the correction of amplification effects, SNP-level raw log2 ratios of the amplified samples were subtracted by the median log2 ratios of the corresponding probe of the 5 amplified normal copy number controls. Raw log2 ratios of each array were then centred to a median of zero. Raw log2 ratios of each sample on both Sty and Nsp arrays were combined and segmented using a faster circular binary segmentation algorithm [67]. Segment means were assigned to genes within the segments for each sample using the CNTools package of Bioconductor (http://www.bioconductor.org). SNP, gene, and cytogenetic band locations are based on the hg18 genome build. Deletion threshold was set to log2 (1.5/2) and amplification threshold to log2 (2.5/2). A gene is considered deleted (amplified) if its assigned log2 ratio is less (greater) than the deletion (amplification) threshold. In the amplified normal samples, less than 0.19% genes have log2 ratio less than the deletion threshold and less than 0.1% genes greater than the amplification threshold.

Expression array data were analysed with GCOS using the default settings except that the target signal was set to 100. Comparisons based on mean expression levels in cancers and controls will fail to detect changes that are restricted to only a few tumours. Therefore the number of tumours in which genes of interest were up-regulated or down-regulated is reported using the following rules. A gene was considered upregulated if its GCOS call in a tumour was “present” and its normalised expression level was greater than twice the mean of the normal samples; and downregulated if it was called “present” in all 4 of the normal samples and its expression level in the tumour was more than twofold less than the mean of the normals. A gene was considered to be unchanged if it met none of these criteria. Application of the above rules to only the normal samples estimated the false positive rate for upregulated genes to be 3.2% whilst that for downregulated genes was 8.7%.

A gene expression heatmap was generated using dChip [66]. Log ratio plots were produced using IGB [68].

An extensive literature search for tumour suppressor genes produced a list of 740 previously identified putative TSGs. A list of 309 potential tumour promoting genes was compiled from genes designated “oncogene” in their NCBI gene title, from the list in [69] and from individual instances in the literature.

### Determination of DNA Copy Number Using Quantitative PCR (q-PCR) Assays

Genomic copy number predicted by the SNP array analysis software was verified by q-PCR using the combinations of forward and reverse primers (Alta Bioscience, University of Birmingham) and Taqman probes (Eurogentec) listed in Table S10. Taqman probes were synthesised containing a 5′ FAM reporter dye and a 3′ TAMRA quencher. All primers and probes were designed using the Primer Express software program (Applied Biosystems).

PCR reactions were prepared in a final volume of 25 µl containing 1x Taqman Universal PCR Mastermix (Applied Biosystems), 300 nM forward and reverse primers, 100 nM probe and either 0.5 ng or 5 ng input DNA. Amplification and detection were performed using an ABI Prism 7500 Sequence Detection System (Applied Biosystems). Thermal cycling conditions comprised an initial, uracil-N glycosylase incubation (2 min, 50°C), AmpliTaq Gold activation step (10 min, 95°C) and 40 rounds of amplification (denaturation for 15 s at 95°C, annealing and extension for 1 min at 60°C). All test samples were run in duplicate and template-negative reactions served as controls. Each experiment also contained serial dilutions of a calibrator DNA derived from the X50-7 cell line corresponding to 10, 10^2^ 10^3^, 10^4^ and 10^5^ gene copies (based on 1 diploid cell being equivalent to 6.6 pg DNA), which were used to generate a standard curve for each target gene.

Real time changes in fluorescence were analysed by the SDS v1.7 software program (Applied Biosystems) and used to determine the Ct value for each sample at which the fluorescence exceeded a threshold value. For each gene, the Ct values for the serial dilutions of the calibrator DNA were used to construct a standard curve from which the copy number of the unknown samples could be extrapolated. To determine if a target sequence was amplified or deleted, each copy number value was then normalised to the reference sequence (CYP7A1).

### Array Data Deposition

The array data associated with this work have been deposited in the GEO database (http://www.ncbi.nlm.nih.gov/geo) with accession number GSE34573.

## Supporting Information

Figure S1 **Expression of EBV genes and EBV genome status of samples used in this study.** The expression of the EBV-encoded genes BARF1, LMP1, LMP2 and EBNA1 was determined by RT-PCR using the primers listed in Table S8. Products were separated by agarose gel electrophoresis and visualised under U.V. light after staining with ethidium bromide. Cellular GAPDH expression was used as a positive control. The primers used in the detection of LMP1 transcripts flank the 33 base pair repeat region. Thus the variation in size of product is due to the different numbers of repeats in the LMP1 coding sequence in the various viral genomes. EBV gene expression in samples YH7 and YH8 was determined in separate experiments (not shown). EBV genome status was determined by PCR as described in the Methods. Samples HK4 and C666-1 were examined separately (not shown).
(TIF)

Table S1 ***A priori*** **defined, putative tumour-related genes upregulated in at least 25% of samples.** X  =  copy number data not applicable on the X chromosome. *NA  =  not available. U  =  upregulated in other cancers. D  =  downregulated in other cancers.
(XLS)

Table S2 ***A priori*** **defined, putative tumour-related genes downregulated in at least 25% of samples.** X  =  copy number data not applicable on the X chromosome. *NA  =  not available. U  =  upregulated in other cancers. D  =  downregulated in other cancers.
(XLS)

Table S3 **TPGs are not enriched with increasing frequency of genomic gain.**
(DOC)

Table S4 **Deletion peaks identified by GISTIC analysis.**
(XLS)

Table S5 **Gain peaks identified by GISTIC analysis.**
(XLS)

Table S6 **Genomic q-pcr validation of SNP array-predicted copy number.** The numbers represent the genome copy number determined by SNP array analysis and by q-pcr.
(XLS)

Table S7 **Proportion of TSGs within homozygous deletions.**
(DOC)

Table S8 **Primers used for RT-PCR detection of EBV-specific latent gene transcripts.** The GAPDH gene was used as a positive control. The LMP1-specific primers flank the 33 base pair repeat region within the coding sequence. The pcr product size is therefore variable due to the different numbers of repeat units found in different virus strains.
(DOC)

Table S9 **Antibodies Used for IHC.**
(DOC)

Table S10 **Primers and probes used for q-PCR.**
(DOC)

## References

[1] Nicholls JM (1997). Nasopharyngeal Carcinoma: Classification and histologic appearances.. Advances in Anatomic Pathology.

[2] Chang ET, Adami HO (2006). The enigmatic epidemiology of nasopharyngeal carcinoma.. Cancer Epidemiol Biomarkers Prev.

[3] Poirier S, Ohshima H, de-Thé G, Hubert A, Bourgade MC (1987). Volatile nitrosamine levels in common foods from Tunisia, south China and Greenland, high-risk areas for nasopharyngeal carcinoma (NPC).. Int J Cancer.

[4] Zheng YM, Tuppin P, Hubert A, Jeannel D, Pan YJ (1994). Environmental and dietary risk factors for nasopharyngeal carcinoma: a case-control study in Zangwu County, Guangxi, China.. Br J Cancer.

[5] Young LS, Rickinson AB (2004). Epstein-Barr virus: 40 years on. Nature Rev.. Cancer.

[6] Li X, Wang E, Zhao Y-D, Ren J-Q, Jin P (2006). Chromosomal imbalances in nasopharyngeal carcinoma: a meta analysis of comparative genomic hybridization results. J. Translat.. Med.

[7] Lo KW, Chung GT, To KF (2012). Deciphering the molecular genetic basis of NPC through molecular, cytogenetic, and epigenetic approaches.. Semin Cancer Biol.

[8] Sriuranpong V, Mutirangura A, Gillespie JW, Patel V, Amornphimoltham P (2004). Global gene expression profile of nasopharyngeal carcinoma by laser capture microdissection and complementary DNA microarrays.. Clin Cancer Res.

[9] Dodd LE, Sengupta S, Chen IH, den Boon JA, Cheng YJ (2006). Genes involved in DNA repair and nitrosamine metabolism and those located on chromosome 14q32 are dysregulated in nasopharyngeal carcinoma.. Cancer Epidemiol Biomarkers Prev.

[10] Sengupta S, den Boon JA, Chen IH, Newton MA, Dahl DB (2006). Genome-wide expression profiling reveals EBV-associated inhibition of MHC class I expression in nasopharyngeal carcinoma.. Cancer Res.

[11] Shi W, Bastianutto C, Li A, Perez-Ordonez B, Ng R (2006). Multiple dysregulated pathways in nasopharyngeal carcinoma revealed by gene expression profiling.. Int J Cancer.

[12] Zeng ZY, Zhou YH, Zhang WL, Xiong W, Fan SQ (2007). Gene expression profiling of nasopharyngeal carcinoma reveals the abnormally regulated Wnt signaling pathway.. Hum Pathol.

[13] Bose S, Yap LF, Fung M, Starzcynski J, Saleh A (2009). The ATM tumour suppressor gene is down-regulated in EBV-associated nasopharyngeal carcinoma.. J Pathol.

[14] Fang W, Li X, Jiang Q, Liu Z, Yang H (2008). Transcriptional patterns, biomarkers and pathways characterizing nasopharyngeal carcinoma of Southern China. J Transl Med..

[15] Zhang W, Zeng Z, Zhou Y, Xiong W, Fan S (2009). Identification of aberrant cell cycle regulation in Epstein-Barr virus-associated nasopharyngeal carcinoma by cDNA microarray and gene set enrichment analysis.. Acta Biochim Biophys Sin.

[16] Strockbine LD, Cohen JI, Farrah T, Lyman SD, Wagener F (1998). The Epstein-Barr virus BARF1 gene encodes a novel, soluble colony-stimulating factor-1 receptor. J Virol..

[17] Decaussin G, Sbih-Lammali F, de Turenne-Tessier M, Bouguermouh A, Ooka T (2000). Expression of *BARF1* gene encoded by Epstein–Barr virus in nasopharyngeal carcinoma biopsies. Cancer Res..

[18] Scholle F, Bendt K M, Raab-Traub N (2000). Epstein–Barr virus LMP2A transforms epithelial cells, inhibits cell differentiation, and activates Akt. J. Virol..

[19] Seto E, Ooka T, Middeldorp J, Takada K (2008). Reconstitution of nasopharyngeal carcinoma-type EBV infection induces tumorigenicity. Cancer Res..

[20] Chow LS, Lo KW, Kwong J, To KF, Tsang KS (2004). RASSF1A is a target tumor suppressor from 3p21.3 in nasopharyngeal carcinoma.. Int J Cancer.

[21] Cheung AK, Lung HL, Hung SC, Law EW, Cheng Y (2008). Functional analysis of a cell cycle-associated, tumor-suppressive gene, protein tyrosine phosphatase receptor type G, in nasopharyngeal carcinoma.. Cancer Res 2008.

[22] Wang GL, Lo KW, Tsang KS, Chung NY, Tsang YS (1999). Inhibiting tumorigenic potential by restoration of p16 in nasopharyngeal carcinoma.. Br J Cancer.

[23] Yau WL, Lung HL, Zabarovsky ER, Lerman MI, Sham JS (2006). Functional studies of the chromosome 3p21.3 candidate tumor suppressor gene BLU/ZMYND10 in nasopharyngeal carcinoma.. Int J Cancer.

[24] Xian J, Aitchison A, Bobrow L, Corbett G, Pannell R (2004). Targeted disruption of the 3p12 gene, Dutt1/Robo1, predisposes mice to lung adenocarcinomas and lymphomas with methylation of the gene promoter.. Cancer Res.

[25] Murakami H, Mizuno T, Taniguchi T, Fujii M, Ishiguro F (2011). LATS2 is a tumor suppressor gene of malignant mesothelioma.. Cancer Res.

[26] Mok SC, Chan WY, Wong KK, Muto MG, Berkowitz RS (1996). SPARC, an extracellular matrix protein with tumor-suppressing activity in human ovarian epithelial cells.. Oncogene.

[27] Alajez NM, Lenarduzzi M, Ito E, Hui AB, Shi W (2011). MiR-218 suppresses nasopharyngeal cancer progression through downregulation of survivin and the SLIT2-ROBO1 pathway. Cancer Res..

[28] Zhang Y, Hu CF, Chen J, Yan LX, Zeng YX (2010). LATS2 is de-methylated and overexpressed in nasopharyngeal carcinoma and predicts poor prognosis. BMC Cancer..

[29] Wang HY, Li YY, Shao Q, Hou JH, Wang F (2012). Secreted protein acidic and rich in cysteine (SPARC) is associated with nasopharyngeal carcinoma metastasis and poor prognosis. J Transl Med..

[30] Ferguson S, Gautrey HE, Strathdee G (2011). The dual role of HLXB9 in leukemia. Pediatr Blood Cancer..

[31] Myal Y, Leygue E, Blanchard AA (2010). Claudin 1 in breast tumorigenesis: revelation of a possible novel “claudin high” subset of breast cancers.. J Biomed Biotechnol.

[32] Bhoumik A, Ronai Z (2008). ATF2: a transcription factor that elicits oncogenic or tumor suppressor activities.. Cell Cycle.

[33] Fang Y, Nicholl MB (2011). Sirtuin 1 in malignant transformation: friend or foe? Cancer Lett..

[34] Abaan OD, Toretsky JA (2008). PTPL1: a large phosphatase with a split personality. Cancer Metastasis Rev..

[35] Johnson DG (2000). The paradox of E2F1: oncogene and tumor suppressor gene. Mol Carcinog..

[36] Mazzarelli P, Pucci S, Spagnoli LG (2009). CLU and colon cancer. The dual face of CLU: from normal to malignant phenotype. Adv Cancer Res..

[37] Bolignano D, Donato V, Lacquaniti A, Fazio MR, Bono C (2010). Neutrophil gelatinase-associated lipocalin (NGAL) in human neoplasias: a new protein enters the scene. Cancer Lett..

[38] Rowland BD, Peeper DS (2006). KLF4, p21 and context-dependent opposing forces in cancer.. Nat Rev Cancer.

[39] Mori A, Moser C, Lang SA, Hackl C, Gottfried E (2009). Up-regulation of Krüppel-like factor 5 in pancreatic cancer is promoted by interleukin-1beta signaling and hypoxia-inducible factor-1alpha.. Mol Cancer Res.

[40] Andreoli V, Gehrau RC, Bocco JL (2010). Biology of Krüppel-like factor 6 transcriptional regulator in cell life and death.. IUBMB Life.

[41] Mangia A, Chiriatti A, Bellizzi A, Malfettone A, Stea B (2009). Biological role of NHERF1 protein expression in breast cancer.. Histopathology.

[42] Massagué J (2008). TGFβ in cancer.. Cell.

[43] Singha PK, Yeh IT, Venkatachalam MA, Saikumar P (2010). Transforming growth factor-beta (TGF-beta)-inducible gene TMEPAI converts TGF-beta from a tumor suppressor to a tumor promoter in breast cancer. Cancer Res..

[44] McDonald SL, Silver A (2009). The opposing roles of Wnt-5a in cancer. Br J Cancer..

[45] Xie LQ, Bian LJ, Li Z, Li Y, Li ZX (2010). Altered expression of E-cadherin by hepatocyte growth factor and effect on the prognosis of nasopharyngeal carcinoma.. Ann Surg Oncol.

[46] Or YY, Chung GT, To KF, Chow C, Choy KW (2010). Identification of a novel 12p13.3 amplicon in nasopharyngeal carcinoma.. J Pathol.

[47] Hui AB, Or YY, Takano H, Tsang RK, To KF (2005). Array-based comparative genomic hybridization analysis identified cyclin D1 as a target oncogene at 11q13.3 in nasopharyngeal carcinoma.. Cancer Res.

[48] Beroukhim R, Getz G, Nghiemphu L, Barretina J, Hsueh T (2007). Assessing the significance of chromosomal aberrations in cancer: methodology and application to glioma. Proc Natl Acad Sci U S A..

[49] Zhao X, Weir BA, LaFramboise T, Lin M, Beroukhim R (2005). Homozygous deletions and chromosome amplifications in human lung carcinomas revealed by single nucleotide polymorphism array analysis.. Cancer Res.

[50] Ko JY, Lee TC, Hsia CF, Lin GL, Yen SH (2002). Definition of three minimal deleted regions by comprehensive allelotyping and mutational screening of FHIT, p16(INK4A), and p19(ARF) genes in nasopharyngeal carcinoma.. Cancer.

[51] Huang DP, Lo KW, van Hasselt CA, Woo JK, Choi PH (1994). A region of homozygous deletion on chromosome 9p21–22 in primary nasopharyngeal carcinoma.. Cancer Res 1994.

[52] Zanesi N, Pekarsky Y, Croce CM (2005). A mouse model of the fragile gene FHIT: From carcinogenesis to gene therapy and cancer prevention.. Mutat Res.

[53] Lo KW, Huang DP (2002). Genetic and epigenetic changes in nasopharyngeal carcinoma.. Semin Cancer Biol.

[54] Chan ASC, To KF, Lo KW, Mak KF, Pak W (2000). High frequency of chromosome 3p deletion in histologically normal nasopharyngeal epithelia from southern Chinese.. Cancer Res.

[55] Gelsi-Boyer V, Orsetti B, Cervera N, Finetti P, Sircoulomb F (2005). Comprehensive profiling of 8p11–12 amplification in breast cancer.. Mol Cancer Res.

[56] Kotliarov Y, Kotliarova S, Charong N, Li A, Walling J (2009). Correlation analysis between single-nucleotide polymorphism and expression arrays in gliomas identifies potentially relevant target genes.. Cancer Res.

[57] Daniel FI, Cherubini K, Yurgel LS, de Figueiredo MA, Salum FG (2011). The role of epigenetic transcription repression and DNA methyltransferases in cancer. Cancer..

[58] Kouzarides Y (2011). Chromatin modifications and their function.. Cell.

[59] Hill CE, Harris SB, Culler EE, Zimring JC, Nolte FS (2006). Performance characteristics of two real-time PCR assays for the quantification of Epstein-Barr virus DNA.. Am J Clin Pathol.

[60] Owen TJ, O’Neil JD, Dawson CW, Hu C, Chen X (2010). Epstein-Barr virus encoded EBNA1 enhances RNA polymerase III-dependent EBER expression through induction of EBER-associated cellular transcription factors.. Mol Cancer.

[61] Li YH, Hu CF, Shao Q, Huang MY, Hou JH (2008). Elevated expressions of survivin and VEGF protein are strong independent predictors of survival in advanced nasopharyngeal carcinoma.. J Transl Med.

[62] Reynolds GM, Deshmukh NS, Mangham DC (2000). Agitated low temperature epitope retrieval (ALTER): effective antigen retrieval for immunohistochemistry with excellent morphological preservation.. J. Pathol.

[63] Cheung ST, Huang DP, Hui ABY, Lo KW, Tsang YS (1999). Nasopharyngeal carcinoma cell line (C666-1) consistently harbouring Epstein-Barr Virus. Int.. J. Cancer.

[64] Wilson G, Miller G (1979). Recovery of Epstein-Barr virus from nonproducer neonatal human lymphoid cell transformants.. Virology.

[65] Morris M, Dawson CW, Wei W, O’Neil JD, Stewart S (2008). The Epstein-Barr virus (EBV)-encoded LMP1 induces a hyperproliferative and inflammatory gene expression programme in cultured keratinocytes. J. Gen.. Virol.

[66] Li C, Wong WH (2001). Model-based analysis of oligonucleotide arrays: Expression index computation and outlier detection. Proc. Natl. Acad. Sci.. U S A.

[67] Venkatraman ES, Olshen AB (2007). A faster circular binary segmentation algorithm for the analysis of array CGH data.. Bioinformatics.

[68] Nicol JW, Helt GA, Blanchard SG, Raja A, Loraine AE (2009). The Integrated Genome Browser: free software for distribution and exploration of genome-scale datasets. Bioinformatics..

[69] Lockwood WW, Chari R, Coe BP, Girard L, Macaulay C (2008). DNA amplification is a ubiquitous mechanism of oncogene activation in lung and other cancers.. Oncogene.

