# Supplementary figures and images for: A Global View of the Oncogenic Landscape in Nasopharyngeal Carcinoma: An Integrated Analysis at the Genetic and Expression Levels
Source: PLoS One. 2012 Jul 17;7(7):e41055. doi: 10.1371/journal.pone.0041055 (PMC3398876; doi:10.1371/journal.pone.0041055)

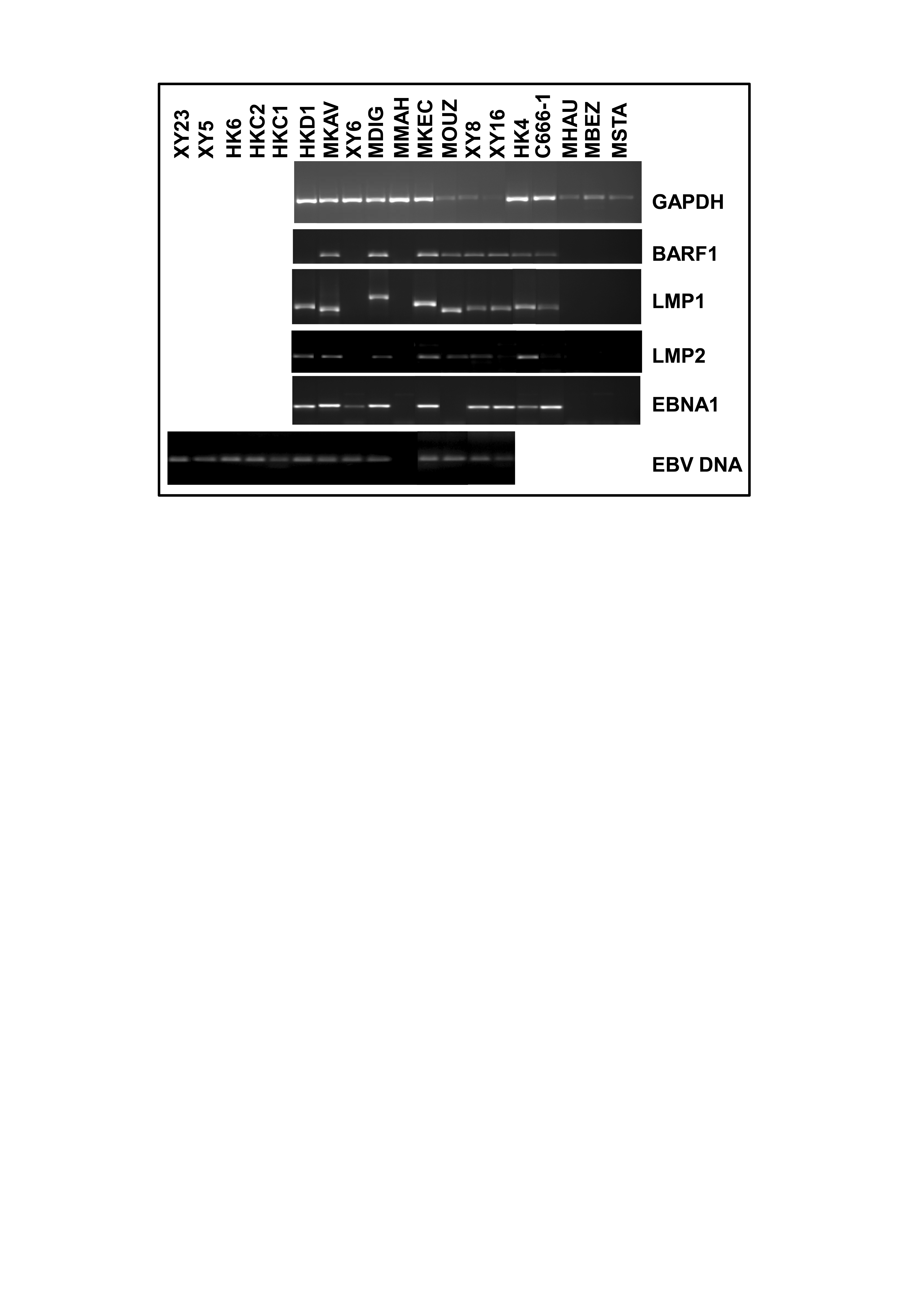

Supplement: Figure S1 — Expression of EBV genes and EBV genome status of samples used in this study. The expression of the EBV-encoded genes BARF1, LMP1, LMP2 and EBNA1 was determined by RT-PCR using the primers listed in Table S8. Products were separated by agarose gel electrophoresis and visualised under U.V. light after staining with ethidium bromide. Cellular GAPDH expression was used as a positive control. The primers used in the detection of LMP1 transcripts flank the 33 base pair repeat region. Thus the variation in size of product is due to the different numbers of repeats in the LMP1 coding sequence in the various viral genomes. EBV gene expression in samples YH7 and YH8 was determined in separate experiments (not shown). EBV genome status was determined by PCR as described in the Methods. Samples HK4 and C666-1 were examined separately (not shown). (TIF) [file pone.0041055.s001.tif]
