# Supplementary material for: A Global View of the Oncogenic Landscape in Nasopharyngeal Carcinoma: An Integrated Analysis at the Genetic and Expression Levels
Source: PLoS One. 2012 Jul 17;7(7):e41055. doi: 10.1371/journal.pone.0041055 (PMC3398876; doi:10.1371/journal.pone.0041055)
Supplement: Table S3 — TPGs are not enriched with increasing frequency of genomic gain. (DOC) [file pone.0041055.s004.doc]

| Number of samples with copy gain in common | Number of amplified genes | Number of amplified TPGs | Percentage of TPGs in amplified genes | P value of binomial test |
| --- | --- | --- | --- | --- |
| 5 or more | 1036 | 14 | 1.35% | 0.2355 |
| 6 or more | 744 | 10 | 1.34% | 0.2871 |
| 7 or more | 424 | 7 | 1.65% | 0.1782 |
| 8 or more | 232 | 1 | 0.43% | 0.9195 |

**Table S3**
