# Supplementary material for: A Global View of the Oncogenic Landscape in Nasopharyngeal Carcinoma: An Integrated Analysis at the Genetic and Expression Levels
Source: PLoS One. 2012 Jul 17;7(7):e41055. doi: 10.1371/journal.pone.0041055 (PMC3398876; doi:10.1371/journal.pone.0041055)
Supplement: Table S7 — Proportion of TSGs within homozygous deletions. (DOC) [file pone.0041055.s008.doc]

**Table S7**

| Number of samples with homozygous deletion | Number of homozygously deleted genes | Number of homozygously deleted TSGs | Percentage of TSGs in homozygously deleted genes | P value of binomial test |
| --- | --- | --- | --- | --- |
| 1 or more | 205 | 24 | 11.7 | 1.47x10^-7^ |
| 2 or more | 69 | 6 | 8.7 | 0.029 |
| 3 or more | 24 | 3 | 12.5 | 0.046 |
| 4 or more | 10 | 0 | 0 | 1 |
