# Supplementary material for: A Global View of the Oncogenic Landscape in Nasopharyngeal Carcinoma: An Integrated Analysis at the Genetic and Expression Levels
Source: PLoS One. 2012 Jul 17;7(7):e41055. doi: 10.1371/journal.pone.0041055 (PMC3398876; doi:10.1371/journal.pone.0041055)
Supplement: Table S8 — Primers used for RT-PCR detection of EBV-specific latent gene transcripts. The GAPDH gene was used as a positive control. The LMP1-specific primers flank the 33 base pair repeat region within the coding sequence. The pcr product size is therefore variable due to the different numbers of repeat units found in different virus strains. (DOC) [file pone.0041055.s009.doc]

| **Gene** | **Primer oligonucleotides (5’-3’)** | **Product size (base pairs)** |
| --- | --- | --- |
| GAPDH | Forward: GCCTCCTGCACCACCAACTG  Reverse: CGACGCCTGCTTCACCACCTTCT | 351 |
| EBNA1 | Forward: TATGACAAAGCCCGCTCCTAC  Reverse: TCACCCTCATCTCCATCACC | 162 |
| LMP1 | Forward: CCCCCACTCTGCTCTCAAAA  Reverse: CCGTGGGGGTCGTCATCAT | Variable around 485 |
| LMP2 | Forward: TGCTGCTACTACTGCCTTAC  Reverse: TGAACAGAGCCCTGCAATG | 185 |
| BARF1 | Forward: GGCTGTCACCGCTTTCTTGG  Reverse: AGGTGTTGGCACTTCTGTGG | 203 |

**Table S8**
