# Supplementary material for: A Global View of the Oncogenic Landscape in Nasopharyngeal Carcinoma: An Integrated Analysis at the Genetic and Expression Levels
Source: PLoS One. 2012 Jul 17;7(7):e41055. doi: 10.1371/journal.pone.0041055 (PMC3398876; doi:10.1371/journal.pone.0041055)
Supplement: Table S9 — Antibodies Used for IHC. (DOC) [file pone.0041055.s010.doc]

**Table S9**

| **Primary antibody** | **Species** | **Dilution** | **Supplier** |
| --- | --- | --- | --- |
| MSH3(S-16) | Goat | 1:50 | Santa Cruz |
| TGFBI(HPA008612) | Rabbit | 1:150 | Sigma |
| ITGA2(CD49b,611016)) | Mouse | 1:100 | Becton Dickinson |
| CLU | Mouse | 1:6000 | Santa Cruz |
| EZH2 | Mouse | 1:200 | Cell Signalling |
| JAK1(sc-1677) | Mouse | 1:100 | Santa Cruz |
| SKIL(HPA013920) | Rabbit | 1:800 | Sigma |
| WNT5A | Rabbit | 1:50 | Santa Cruz |
| KLF4(AF3640) | Goat | 1:100 | R&D Systems |
| LCN2(HPA002695) | Rabbit | 1:100 | Sigma |
| ANXA1(HPA011271) | Rabbit | 1:800 | Sigma |
| TNFAIP3(HPA002116) | Rabbit | 1:20 | Sigma |
| CD44 (v6) | Mouse | 1:200 | R&D Systems |
| CTNNB1(610153) | Rabbit | 1:400 | Becton Dickinson |
