# Supplementary material for: A Global View of the Oncogenic Landscape in Nasopharyngeal Carcinoma: An Integrated Analysis at the Genetic and Expression Levels
Source: PLoS One. 2012 Jul 17;7(7):e41055. doi: 10.1371/journal.pone.0041055 (PMC3398876; doi:10.1371/journal.pone.0041055)
Supplement: Table S10 — Primers and probes used for q-PCR. (DOC) [file pone.0041055.s011.doc]

**Table S10**

| **Gene** | **Primer and Probe Sequences 5’-3’** |
| --- | --- |
| FHIT | Primer Forward: TGGGAGGGAGATGGATTCTTTA  Reverse: CTTCTGAGCCTAACCAGCCAAA  Probe: TGGACAGACTGGAGGCCAATCTTGTATTTATATTC |
| MAP3K5 | Primer Forward: GTCAATGATAGCCTTCCACAGTGT  Reverse: GAAATGTTCGTTTCACCATGTTCT  Probe: CACAGCATCCCTCCCCTGTTTAAAGACA |
| CDKN2A | Primer Forward: GCGTGAGCTGAGGCAAGAC  Reverse: TCCAAAGCTCAGAGCATTCATTT  Probe: CTGGTCTCCCGGGCTGAACTTTCTG |
| IKBKB | Primer Forward: TTGCTTATAGAGTTAGCACGACATCA  Reverse: TCCCAGGCCCCACATG  Probe: ATGAGCTGGTCACCTTCCCTGACAACG |
| CYP7A1 | Primer Forward: TGATATGTTGGTGGAAAGGATTACTAA  Reverse: TAGACGGGTGCTTGTTGAATGT  Probe: TCCATCCATTCATGCATCCGTCCATA |
